# Transcriptome Sequencing Identifies *PLAUR* as an Important Player in Patients With Dermatomyositis-Associated Interstitial Lung Disease

**DOI:** 10.3389/fgene.2021.784215

**Published:** 2021-12-06

**Authors:** Juan Chen, Ruixian Zhang, Min Xie, Chunyan Luan, Xiaolan Li

**Affiliations:** ^1^ Department of Rheumatology and Clinical Immunology, The Second Affiliated Hospital of Kunming Medical University, Kunming, China; ^2^ The Center for Disease Control and Prevention of Yunnan Province, Kunming, China; ^3^ Department of Dermatology and Venereology, The Second Affiliated Hospital of Kunming Medical University, Kunming, China

**Keywords:** dermatomyositis, interstitial lung disease, RNA-sequencing, alternative splicing, plasminogen activator, urokinase receptor

## Abstract

Dermatomyositis (DM), an inflammatory disorder, is often associated with interstitial lung disease (ILD). However, the underlying mechanism remains unclear. Our study performed RNA sequencing (RNA-seq) and integrative bioinformatics analysis of differentially expressed genes (DEGs) in patients with dermatomyositis-associated interstitial lung disease (DM-ILD) and healthy controls. A total of 2,018 DEGs were identified between DM-ILD and healthy blood samples. Gene Ontology (GO) and Kyoto Encyclopedia of Genes and Genomes (KEGG) pathway enrichment analysis showed that DEGs were mainly involved in immune- and inflammatory-related biological processes and pathways. Disease ontology (DO) enrichment analysis identified 35 candidate key genes involved in both skin and lung diseases. Meanwhile, a total of 886 differentially expressed alternative splicing (AS) events were found between DM-ILD and healthy blood samples. After overlapping DEGs with differential AS genes, the plasminogen activator and urokinase receptor (*PLAUR*) involved in immune-related biological processes and complement and coagulation cascades was screened and identified as the most important gene associated with DM-ILD. The protein–protein interaction (PPI) network revealed that *PLAUR* had interactions with multiple candidate key genes. Moreover, we observed that there were significantly more neutrophils and less naive B cells in DM-ILD samples than in healthy samples. And the expression of *PLAUR* was significantly positively correlated with the abundance of neutrophils. Significant higher abundance of *PLAUR* in DM-ILD patients than healthy controls was validated by RT-qPCR. In conclusion, we identified *PLAUR* as an important player in regulating DM-ILD by neutrophil-associated immune response. These findings enrich our understanding, which may benefit DM-ILD patients.

## Introduction

Dermatomyositis (DM) is an autoimmune disease characterized by the inflammation of skeletal muscles and skin lesions. DM is the most common subtype of idiopathic inflammatory myopathies (Lilleker et al., 2018), with interstitial lung disease (ILD) accounting for most of the mortality (Vuillard et al., 2018; Hervier and Uzunhan, 2019). However, patients with ILD may have minimal, absent, or late-onset myositis or skin lesions (Friedman et al., 1996; Tillie-Leblond et al., 2008). The lack of correlation between myositis and pulmonary symptoms often leads to delayed diagnosis and a compromised therapeutic response. In addition, MHC polymorphisms (Miller et al., 2013), epigenetic modification, and miRNA activity (Gao et al., 2018; Gao et al., 2019), which are important regulators of gene expression, have been reported to play a role in DM pathogenesis. For these reasons, the identification of key molecules involved in DM-ILD is highly required for improving the clinical outcome, and the role of their transcriptional regulations in the etiology of DM has great significance for investigation.

Alternative splicing (AS) is the process by which different splice sites in precursor messenger RNA (mRNA) are selected to generate multiple mRNA isoforms. It is an essential and ubiquitous mechanism for regulating gene expression. AS events increase the diversity of self-antigens in specific tissues and/or different developmental stages and create an additional challenge for the immune system to avoid self-reactivity (Nilsen and Graveley, 2010). AS is also closely related to immune function and autoimmune disease. Activated T cells expressed a γ-chain mRNA splice isoform as an active immunoregulatory molecule, which promotes inflammatory T-cell immune responses *in vivo* (Hong et al., 2014). Exons in autoimmune disease and type 1 diabetes candidate genes were more likely to be differentially expressed and alternatively spliced than the expressed exons of other genes (Newman et al., 2017). However, few studies on AS events in DM-ILD have been performed.

Therefore, the present study was designed to explore the role of AS in the pathogenesis of DM-ILD and identify AS-associated key genes involved in DM-ILD *via* RNA-seq.

## Materials and Methods

### Sample Collection and Data Processing

Peripheral blood was obtained from 16 DM-ILD patients and 16 healthy controls. There was no significant difference in the general characteristics between the disease group and the control group (Supplementary Table S1). There was no significant difference in age and clinical data in the DM-ILD patients (Table 1, Table 2). All DM-ILD patients were treated with glucocorticoids and immunosuppressive agents, including prednisone acetate, methylprednisolone, methotrexate, cyclophosphamide, tacrolimus, and cyclosporin A.

**TABLE 1:** Clinical data of the 16 DM-ILD patients.

Patient	MDAAT	ALT (U/L)	AST (U/L)	LDH (U/L)	CK (U/L)	IL-6 (pg/ml)	hsCRP (ng/dl)
D1	6	63	92	452	542	<1.5	3.31
D2	15	37	29	238	23	9.5	0.94
D3	11	41	17	475	29	<1.5	2.88
D4	5	26	18	241	33	3.36	1.62
D5	12	10	12	202	14	27.59	19.7
D6	16	106	122	1,640	2,758	10.72	11.61
D7	5	46	51	236	38	7.24	5.27
D8	4	22	17	240	63	3.78	0.84
D9	18	20	27	174	26	826.2	87.61
D10	15	33	61	415	236	10.39	1.4
D11	4	267	310	1,287	6,584	11.81	4.64
D12	4	8	14	226	22	124.9	7.74
D13	10	63	21	157	29	<1.5	1.45
D14	6	106	82	140	158	10.72	11.61
D15	17	35	37	226	26	12.29	0.59
D16	13	36	61	311	58	5.21	3.48

MDAAT, myositis disease activity assessment tool; ALT, glutamate pyruvate transaminase; AST, glutamic oxaloacetic acid transferase; LDH, lactic dehydrogenase; CK, creatine kinase; IL, interleukin; hsCRP, high-sensitivity C-reactive protein.

**TABLE 2:** Features of included patients.

Variable	DM-ILD patients		
	RNA sequencing (n = 6)	RT-qPCR (n = 10)	*p* value
Age (years)	48.3 ± 8.4	52.2 ± 10.4	0.45
MDAAT	10.80 ± 4.50	9.60 ± 5.70	0.66
ALT	47.17 ± 33.71	35.50 (21.50, 73.75)	0.88
AST	23.50 (15.75, 99.50)	44.00 (20.00, 66.25)	0.64
LDH	346.50 (229.00, 766.25)	231.00 (169.75, 337.00)	0.15
CK	31.00 (20.75, 1,096.00)	48.00 (26.00, 177.50)	0.71
IL-6	8.86 ± 10.08	10.56 (4.85, 40.44)	0.26
hsCRP	6.67 ± 7.46	4.06 (1.26, 8.70)	0.88
Anti-MDA5 antibody	3/6	4/10	
Anti-SRP antibody	1/6	2/10	
Anti-NXP-2 antibody	1/6	0	
Anti-Mi-2 antibody	1/6	0	
Anti-PL-7 antibody	0	1/10	
Anti-PL-12 antibody	0	1/10	
Anti-SAE-1 antibody	0	1/10	
Anti-JO-1antibody	0	1/10	
Anti-RO-52 antibody	4/6	9/10	

Whole blood samples were collected from six DM-ILD patients (ID: LR20L26D X196, LR20L26DX197, LR20L26DX198, LR20L26DX199, LR20L26DX200, and LR20L26DX201) and six healthy controls (ID: LR20L26DX202, LR20L26DX203, LR20L26DX204, LR20L26DX205, LR20L26DX206, and LR20L26DX207) for RNA- seq. Total RNA was isolated and purified using TRIzol reagent (Invitrogen, Carlsbad, CA, United States) following the manufacturer’s protocol. The RNA amount and purity of each sample were quantified using NanoDrop ND-1000 (NanoDrop, Wilmington, DE, United States). The RNA integrity was assessed by Bioanalyzer 2100 (Agilent, CA, United States), with a RIN number >7.0, and confirmed by electrophoresis with denaturing agarose gel. Poly(A) RNA is purified from 1 μg total RNA using Dynabeads Oligo (dT)25-61005 (Thermo Fisher, CA, United States) using two rounds of purification. Then the poly(A) RNA was fragmented into small pieces using the Magnesium RNA Fragmentation Module (NEB, Cat. e6150, United States) under 94°C for 5–7 min. Then the cleaved RNA fragments were reverse-transcribed to create the cDNA by SuperScript™ II Reverse Transcriptase (Invitrogen, Cat. 1896649, United States), which were next used to synthesize U-labeled second-stranded DNAs with *E. coli* DNA polymerase I (NEB, Cat. m0209, United States), RNase H (NEB, cat. m0297, United States), and dUTP Solution (Thermo Fisher, Cat. R0133, United States). The A-base is then added to the blunt ends of each strand, preparing them for ligation to the indexed adapters. Each adapter contains a T-base overhang for ligating the adapter to the A-tailed fragmented DNA. Single- or dual-index adapters are ligated to the fragments, and size selection was performed with AMPureXP beads. After the heat-labile UDG enzyme (NEB, Cat. m0280, United States) treatment of the U-labeled second-stranded DNA, the ligated products are amplified with PCR by the following conditions: initial denaturation at 95°C for 3 min; eight cycles of denaturation at 98 C for 15 s, annealing at 60°C for 15 s, extension at 72°C for 30 s; and then final extension at 72°C for 5 min. The average insert size for the final cDNA library was 300 ± 50 bp. At last, we performed the 2 × 150 bp paired-end sequencing (PE150) on an Illumina NovaSeq™ 6000 (LC-Bio Technology CO., Ltd. Hangzhou, China) following the vendor’s recommended protocol. The quality of sequencing data was evaluated by FastQC (version 0.11.9). The raw data were filtered by Trimmomatic (version 0.39). Then clean data were mapped to the reference genome of *Homo sapiens* GRCh38 using HISAT2 (version 2.2.1) with default parameters. Gene counts were calculated by FeatureCount (version 2.0.0). DEGs were identified by R package DESeq2 (version 1.26.0) with | log2 (fold change) | ≥1 and *p*-value < 0.05. AS events were analyzed by RMATS (version 3.1.1), and differentially expressed AS events were screened with FDR ≤0.05 and |IncLevelDifference| ≥ 0.1 (Shen et al., 2012) (Shen et al., 2014). Gene Ontology (GO), Kyoto Encyclopedia of Genes and Genomes (KEGG) pathway, and disease ontology (DO) enrichment analysis were analyzed using R package clusterProfiler. The quantitative analysis of interactive sets between different types of AS was displayed in UpSet plot. The detail of differentially expressed genes (DEGs) and differentially expressed AS events in the chromosome was visualized in the Circos plot.

### Identification of Key Genes and Construction of PPI Network in DM-ILD

After DO analysis, DEGs that were enriched into both skin and lung diseases were selected and identified as candidate key genes. Then by overlapping candidate key genes with parent genes of differentially expressed AS, key genes associated with DM-ILD were identified. Key genes and candidate key genes were input into the STRING database to analyze the interactions, and the PPI network was visualized by Cytoscape software (version 3.6.1).

### Immune Infiltration Analysis of DM-ILD Patients

CIBERSORT was applied to assess the infiltration of 22 immune cells in different subgroups, including naive B cells, memory B cells, plasma cells, CD8 T cells, naive CD4 T cells, resting memory CD4 T cells, activated memory CD4 T cells, follicular helper T cells, regulatory T cells (Tregs), gamma delta T cells, resting NK cells, activated NK cells, monocytes, M0 macrophages, M1 macrophages, M2 macrophages, resting dendritic cells, activated dendritic cells, resting mast cells, activated mast cells, eosinophils, and neutrophils. Then the *t* test was used to compare the difference of immune infiltration between six DM-ILD patients and six healthy controls. The correlations between key genes and differentially distributed immune cells were calculated.

### RT-qPCR

Total RNA of ten DM-ILD patients and ten control subjects were isolated from peripheral blood using TRIzol reagent (15596018, Life Technologies, American). And RNA was reverse-transcribed to cDNA *via* the SureScript-First Strand cDNA Synthesis Kit (QP057, GeneCopoeia, Guangzhou, China). The expression of *PLAUR* was detected by Bio-Rad CFX96 (American) using BlazeTaq™ SYBR^®^ Green qPCR Mix 2.0 (QP033, GeneCopoeia, Guangzhou, China). PCR amplification cycle conditions were 95°C 30 s, 95°C 10 s, 60°C 20 s, and 72°C 30 s for 40 cycles. GAPDH was used as the internal standardized reference. The relative expression level was calculated using the 2^−ΔΔCt^ method. The specific primers were as follows: *PLAUR*, 5′-CGAGGTTGT GTGTGGGTTA-3’ (forward) and 5′-GGC​ACT​GTT​CTT​CAG​GGC​T-3’ (reverse); GAPDH, 5′- CGC​TGA​GTA​CGT​CGT​GGA​GTC-3′ (forward) and 5′- GCTGATGAT CTTGAGGCTGTTGTC-3′ (reverse).

### Statistical Analysis

All data were analyzed by R (version 4.0.0). Student’s t test was used to compare differences between healthy controls and DM-ILD. Pearson’s correlations between the expressions of key genes and abundance of differentially infiltrated immune cells were calculated. *P-*values less than or equal to 0.05 were considered statistically significant.

## Results

### Identification of DEGs Between DM-ILD Patients and Healthy Individuals

**FIGURE 1:**
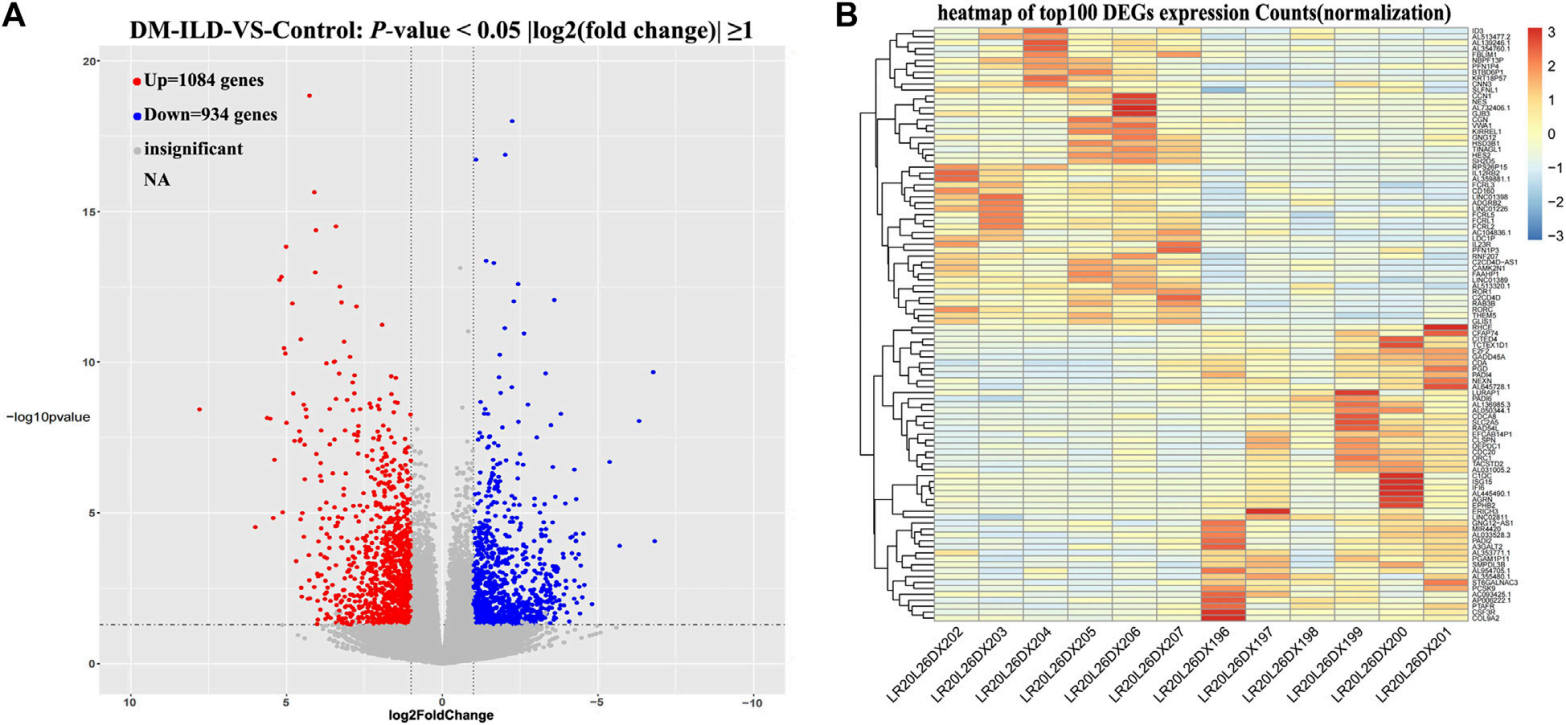
Differentially expressed mRNAs between DM-ILD and control samples. **(A)** 1,084 upregulated and 934 downregulated genes in DM-ILD patients relative to healthy ones were identified using a cutoff as “two-fold expression difference and *p*-value < 0.05.” **(B)** Expressions of top 50 upregulated and top 50 downregulated genes in each sample were displayed in the heatmap.

The quality scores, sequence content, and duplication levels of raw sequencing data were evaluated by FastQC and are shown in Supplementary Material S1. To obtain high-quality data for following analysis, low-quality reads were discarded, and the reads contaminated with adaptor sequences were trimmed using Trimmomatic. Thereafter, quality control of clean data was performed by FastQC and is shown in Supplementary Material S2. Statistical results of raw data and clean data are shown in Supplementary Material S3, proving that clean data can be used for following mapping. Then the clean data were mapped to the GRCh38 reference genome, and we found that the percentage of mapped reads was over 79% (Supplementary Material S4), indicating that the clean data meet the needs for following analyses. Next, gene counts were evaluated by FeatureCount (Supplementary Material S5), and the expressions of genes in each sample were calculated. Principal component analysis by R package ggplot2 showed that samples within the same group had good replication (Supplementary Figure S1). A total of 2,018 DEGs were found, including 1,084 upregulated and 934 downregulated genes in DM-ILD patients relative to healthy ones (Figure 1A). The expressions of top 50 upregulated and top 50 downregulated genes in each sample were displayed in the heatmap (Figure 1B).

### Functional Enrichment of DEGs in DM-ILD

**FIGURE 2:**
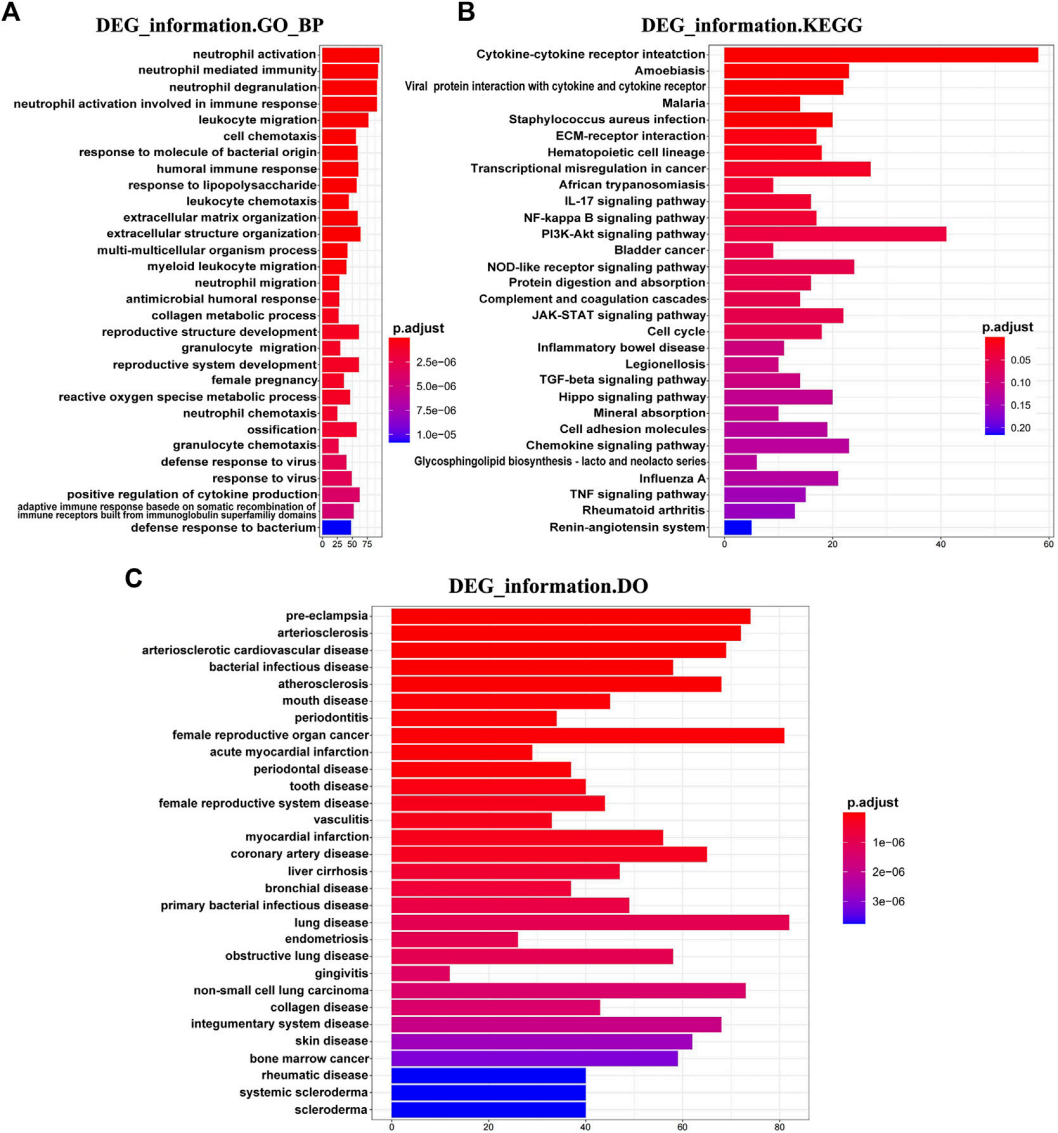
Functional enrichment of DEGs in DM-ILD. **(A)** DEGs were mainly enriched into immune and inflammation biological processes in GO analysis. **(B)** KEGG pathways indicated that DEGs involved in DM-ILD had a relationship with immune and inflammation. **(C)** DEGs were associated with multiple diseases in DO enrichment analysis.

Thereafter, we investigated the biological function of DEGs in DM-ILD. We found that DEGs were mainly enriched into immune and inflammation biological processes, such as neutrophil activation, neutrophil-mediated immunity, neutrophil degranulation, neutrophil activation involved in immune response, leukocyte migration, humoral immune response, leukocyte chemotaxis and response to the molecule of bacterial origin (Figure 2A), and KEGG pathways, such as cytokine–cytokine receptor interaction, ECM-receptor interaction, IL-17 signaling pathway, NF-kappa B signaling pathway, PI3K-Akt signaling pathway, NOD-like receptor signaling pathway, complement and coagulation cascades, and JAK-STAT signaling pathway (Figure 2B). All of these results indicated that DEGs involved in DM-ILD had a strong relationship with immunity and inflammation. To explore the connection of DEGs and diseases, we further performed DO analysis. We found that DEGs were associated with multiple diseases, such as lung disease, skin disease, arteriosclerotic cardiovascular disease, bacterial infectious disease, obstructive lung disease, and female reproductive system disease (Figure 2C).

### Identification of Differentially Expressed AS Events in DM-ILD

**FIGURE 3:**
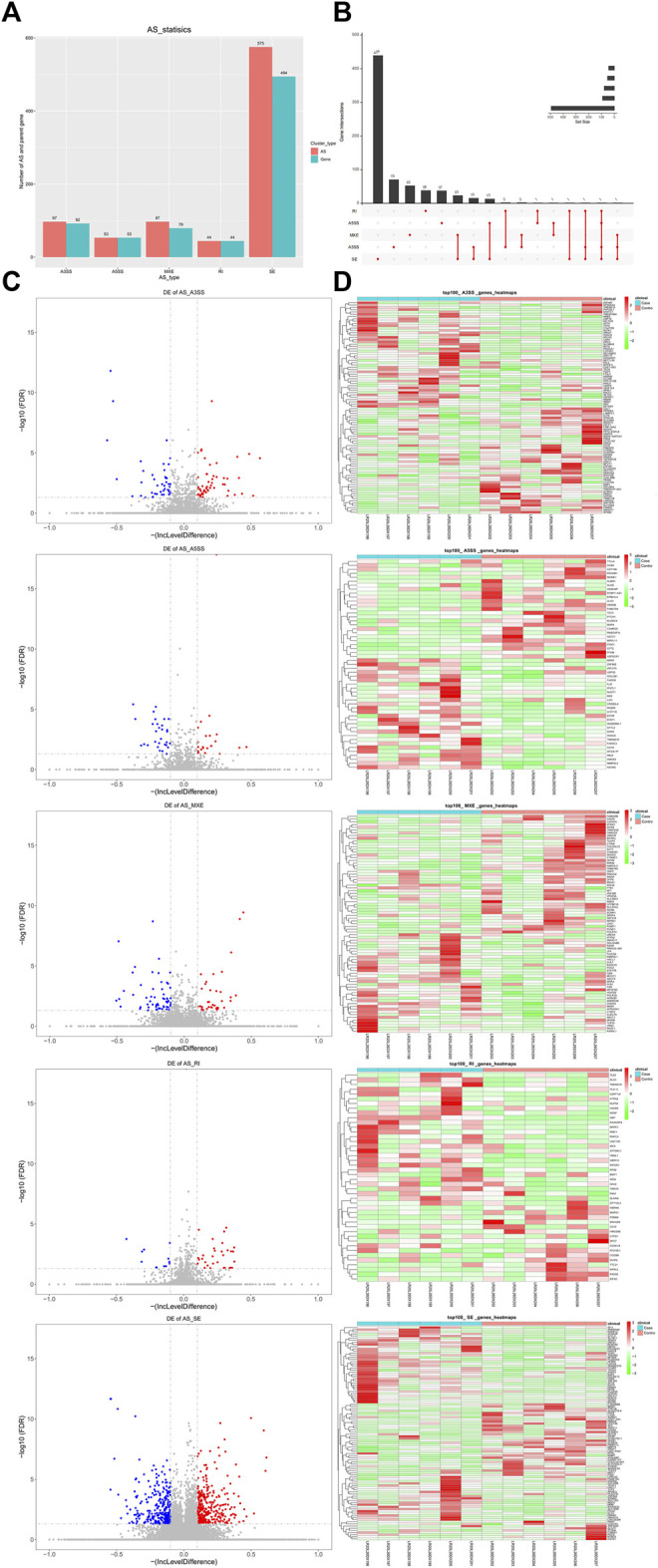
Differentially expressed AS events in between DM-ILD and control samples. **(A)** 866 differentially expressed AS events from 697 genes, including 97 A3SS in 92 genes, 53 A5SS in 53 genes, 97 MXE in 79 genes, 44 RI in 44 genes, and 575 SE in 494 genes were obtained by RMATS and screened with FDR ≤0.05 and |IncLevelDifference| ≥ 0.1. **(B)** Intersecting sets of each AS form. **(C)** Volcano plots showed the upregulated and downregulated AS events of each AS form. **(D)**Top 50 upregulated and top 50 downregulated AS events in each AS type were displayed in the heatmaps.

By analyzing the transcriptome data, five AS forms, exon skipping (SE), alternative 5 and 3′ splice sites (A5SS and A3SS), mutually exclusive exons (MXE), and intron retention (RI) were identified in DM-ILD patients and healthy ones. After comparison, we obtained 866 differentially expressed AS events from 697 genes, including 97 A3SS in 92 genes, 53 A5SS in 53 genes, 97 MXE in 79 genes, 44 RI in 44 genes, and 575 SE in 494 genes (Figure 3A), indicating that one gene had one or more AS events. Therefore, we used UpSet plot to visualize the intersecting sets of each AS form. As shown in Figure 3B, most genes (91.25%) had only one AS type and 61 genes had two to four types of AS events, including a combination of MXE and SE, A3SS and SE, A5SS and SE, RI and A3SS, MXE and A3SS, RI and A5SS, A5SS and MXE, RI and SE, RI, A5SS, A3SS and SE, MXE, and A3SS and SE. The volcano plots shown in Figure 3C showed the upregulated and downregulated AS events of each AS form. Specifically, there were 54 upregulated and 43 downregulated A3SS, 23 upregulated and 30 downregulated A5SS, 308 upregulated and 267 downregulated SE, 32 upregulated and 12 downregulated RI, and 37 upregulated and 60 downregulated MXE events. Top 50 upregulated and top 50 downregulated AS events in each AS type were displayed in the heatmaps (Figure 3D).

### Identification of Key Genes Associated With DM-ILD

**FIGURE 4:**
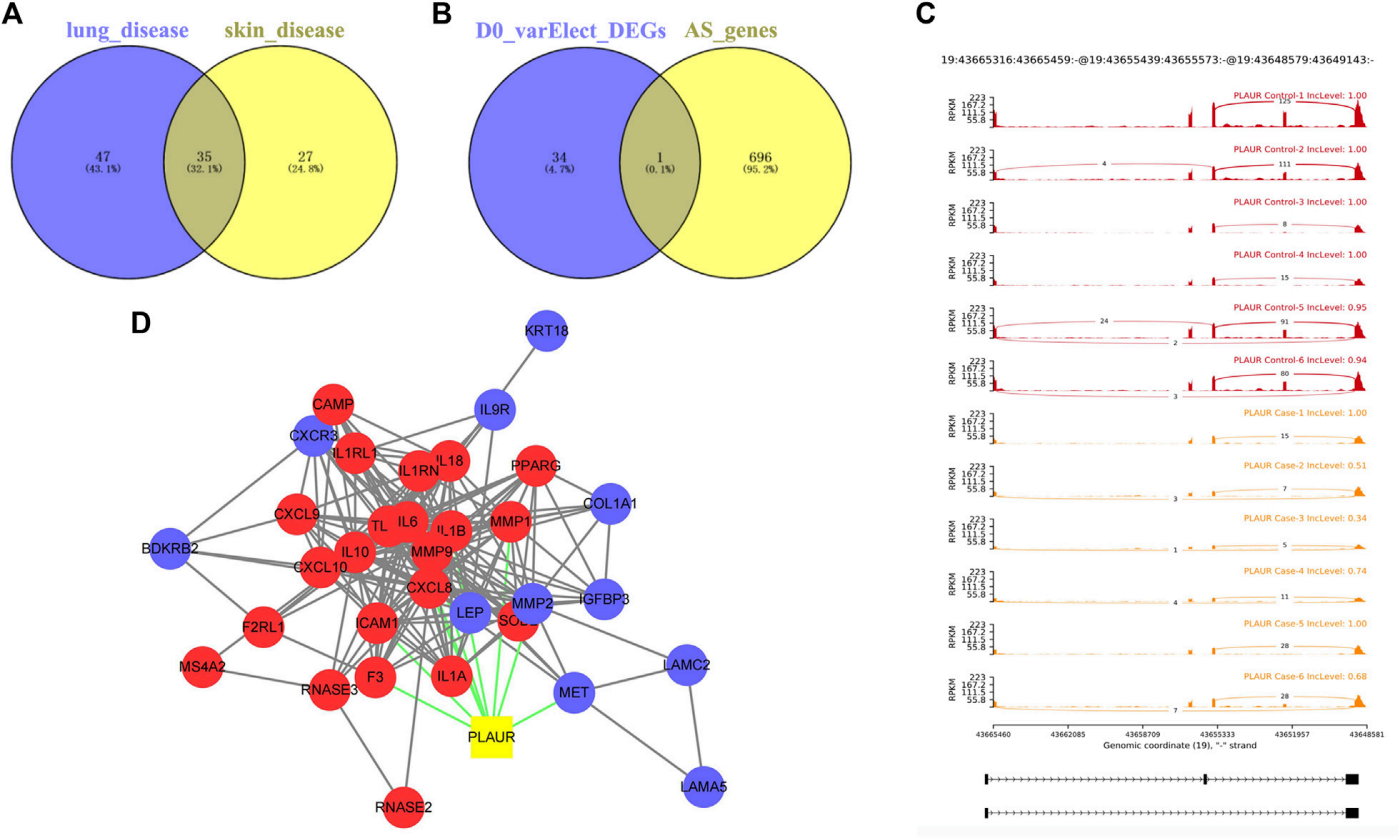
Key genes associated with DM-ILD. **(A)** 35 common genes from lung disease and skin disease in DO analysis. **(B)** *PLAUR* as the key gene in AS-dependent DM-ILD was identified by overlapping 35 genes with the parent genes of differentially expressed AS events. **(C)** *PLAUR* had upregulated SE within chr19:43665316–43665459 in DM-ILD. **(D)** PPI network showed that *PLAUR* had interactions with multiple candidate key genes.

**FIGURE 5:**
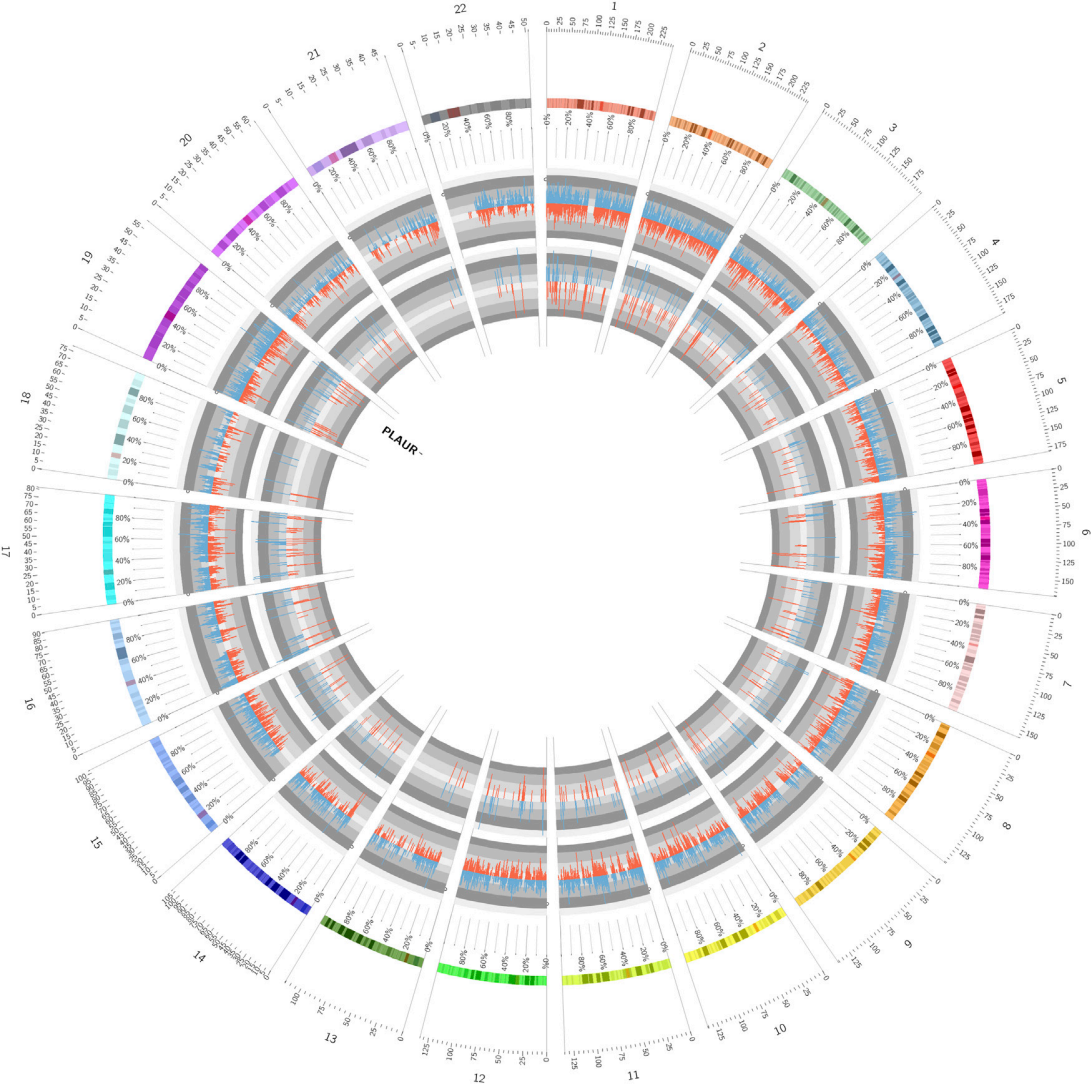
Circos plot showed a full view of DEGs, differentially expressed AS events, and the detail of *PLAUR*.

**FIGURE 6:**
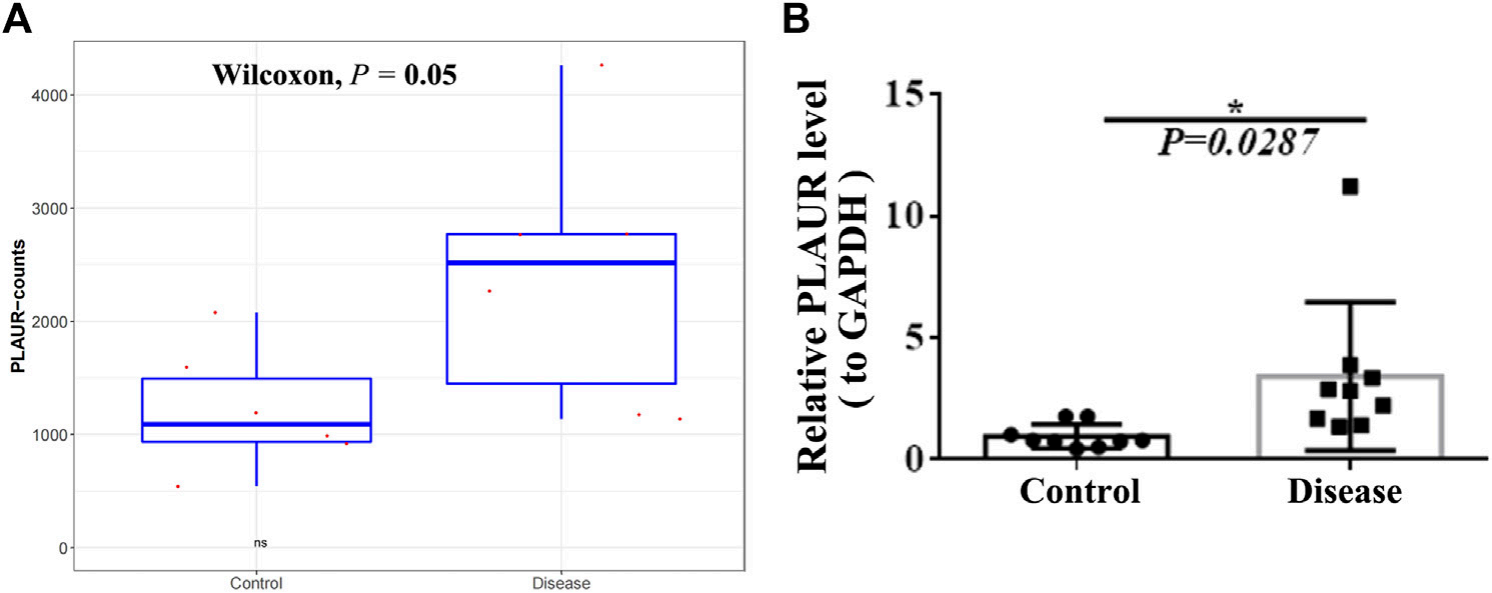
Expression of *PLAUR* in DM-ILD and control samples. **(A)** Abundance of *PLAUR* was higher in DM-ILD samples than control ones in RNA-sequencing. **(B)** Expression of *PLAUR* was significant elevated in the DM-ILD samples by RT-qPCR.

To screen key genes associated with DM-ILD, we extracted 35 common genes from lung disease and skin disease in DO analysis (Figure 4A), namely, *BDKRB2*, *CAMP*, *COL1A1*, *F2RL1*, *F3*, *MS4A2*, *CXCR3*, *ICAM1*, *IGFBP3*, *IGHE*, *IL1A*, *IL1B*, *IL1RN*, *IL6*, *CXCL8*, *IL9R*, *IL10*, *IL18*, *CXCL10*, *KRT18*, *LAMA5*, *LAMC2*, *LEP*, *MET*, *CXCL9*, *MMP1*, *MMP2*, *MMP9*, *PLAUR*, *PPARG*, *RNASE2*, *RNASE3*, *SOD2*, *TLR4*, *and IL1RL1*. Using “Dermatomyositis” or “Pulmonary interstitial fibrosis” or “Inflammation” or “Immunization” as key words on the VarElect database, we found that all of these 35 genes were associated with inflammation and immunization. Thus, these 35 genes were identified as candidate key genes. To further explore the role of AS, we overlapped 35 genes with parent genes of differentially expressed AS events and identified *PLAUR* as the key gene in AS-dependent DM-ILD (Figure 4B). We found that *PLAUR* had upregulated SE within chr19:43665316–43665459 in DM-ILD (Figure 4C). According to GO and KEGG pathway enrichment analyses, *PLAUR* genes were found to participate in complement and coagulation cascades, neutrophil-associated immune response, and coagulation-related biological responses, such as neutrophil activation, neutrophil-mediated immunity, neutrophil degranulation, neutrophil activation involved in immune response, blood coagulation, and negative regulation of coagulation. Next, we constructed the PPI network and found that *PLAUR* had interactions with multiple candidate key genes, including *CXCL8*, *F3*, *ICAM1*, *IL1B*, *IL6*, *MET*, *MMP1*, *MMP2*, and *MMP9* (Figure 4D). Moreover, we generated a Circos plot to give a full view of DEGs, differentially expressed AS events, and the detail of *PLAUR* (Figure 5). By RNA-seq, we found that the abundance of *PLAUR* was higher in DM-ILD samples than the control ones (Figure 6A). Consistent with our sequencing results, RT-qPCR showed that the expression of *PLAUR* was significantly elevated in the DM-ILD samples (Figure 6B).

### Immune Infiltration of DM-ILD Patients

**FIGURE 7:**
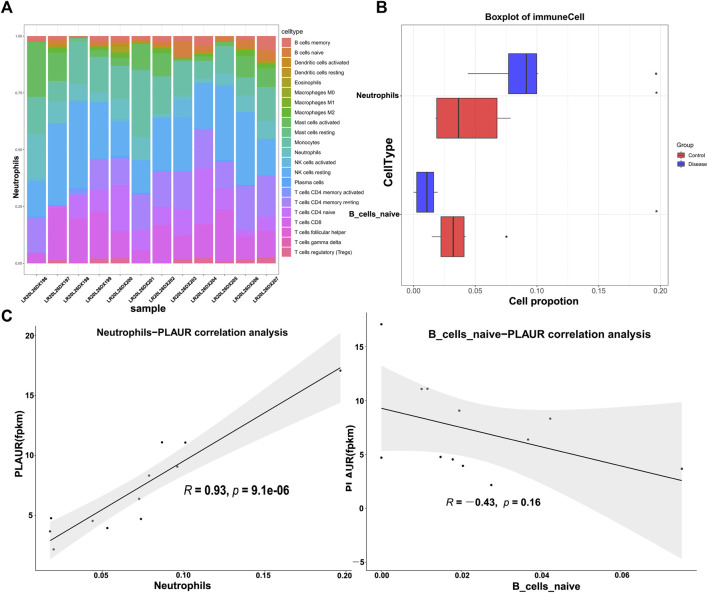
Immune infiltration analysis. **(A)** Abundance of 22 immune cell types in each sample of DM-ILD patients and healthy ones. **(B)** More neutrophils and less naive B cells were detected in DM-ILD patients. **(C)** Correlations between the expression of *PLAUR* and differentially infiltrated immune cells (neutrophils and naive B cells) were displayed in the plot.

DM-ILD was associated with the immune cells. Given the role of immune cells in the immune system, we evaluated the abundance of 22 immune cell types in each sample from DM-ILD patients and healthy ones (Figure 7A). After removing activated memory CD4 T cells, follicular helper T cells, gamma delta T cells, M0 macrophages, M1 macrophages, resting mast cells, and eosinophils with no or few infiltrations in both DM-ILD patients and health ones, the abundance of the remaining 15 immune cell types was compared. We observed that there were significantly more neutrophils and less naive B cells in DM-ILD patients (Figure 7B and Supplementary Figure S2). Given that *PLAUR* was involved in immune-related biological processes and pathways, we calculated the correlations between *PLAUR* and neutrophils or naive B cells. Notably, a strong positive correlation (cor = 0.93) was observed between the expression of *PLAUR* and the abundance of neutrophils (Figure 7C).

## Discussion

Pulmonary involvement is a common complication of DM, causing a significant impact on patient morbidity and mortality. However, only a small number of gene markers in DM-ILD have been explored. In this study, we examined the blood samples in DM-ILD and healthy groups to identify novel candidate biomarkers and biological pathways involved in the etiology of DM.

We identified 2,018 DEGs between DM-ILD patients and healthy individuals. These DEGs were mainly enriched in the immune and inflammation biological processes and pathways. Among them, neutrophil-related biological processes were the ones at the top, with neutrophil activation ranking the first, followed by neutrophil-mediated immunity, neutrophil degranulation, and neutrophil activation involved in immune response. In DM-ILD, a number of different neutrophil disturbances have been described. For instance, the aberrant neutrophil extracellular trap (NET) formation may be involved in the pathogenesis of ILD in DM patients (Peng et al., 2018), and proteinase 3, one type of neutrophil serine proteinases, is lower in patients of DM without ILD (Gao et al., 2018). We also confirmed previous observations related to the pathogenesis of DM and DM-ILD, including the presence of the cytokine–cytokine receptor interaction (Zhu et al., 2017; Chen et al., 2018), IL−17 signaling pathway (Nasr et al., 2012). Additionally, we observed that complement and coagulation cascade pathway may play a critical role in DM-ILD. This pathway has been reported in immune diseases, such as multiple sclerosis (Li et al., 2021), Ig A nephropathy, and Henoch–Schönlein purpura nephritis (Fang et al., 2021). Complement activation has been demonstrated in previous reports (Roy et al., 2020; Tripathi et al., 2021) to be associated with type I interferon, which was significantly activated in DM and DM-ILD (Zhang et al., 2019a; de Jesus et al., 2020). These reports combined with our findings suggest direct and/or indirect links between the two pathways implicated in DM-ILD pathogenesis.

Considering that ILD is one of the severe complications of DM, we focused on 35 genes which were enriched in skin diseases and lung diseases in DO analysis. Consistent with our previous results of functional enrichment, these 35 genes were related to inflammation and immunization. Thus, these 35 genes, namely, *BDKRB2*, *CAMP*, *COL1A1*, *F2RL1*, *F3*, *MS4A2*, *CXCR3*, *ICAM1*, *IGFBP3*, *IGHE*, *IL1A*, *IL1B*, *IL1RN*, *IL6*, *CXCL8*, *IL9R*, *IL10*, *IL18*, *CXCL10*, *KRT18*, *LAMA5*, *LAMC2*, *LEP*, *MET*, *CXCL9*, *MMP1*, *MMP2*, *MMP9*, *PLAUR*, *PPARG*, *RNASE2*, *RNASE3*, *SOD2*, *TLR4*, and *IL1RL1* were identified as candidate key genes. Many studies have characterized that *IL-6*, *IL8*, *IL-18*, and *IL-10* were significantly elevated in DM-ILD and associated with disease activity and prognosis (Gono et al., 2010; Gono et al., 2014; Chen et al., 2018). The important role of *CXCL9* and *CXCL10* in the pathophysiology of DM-ILD has also been reported. The serum levels of *CXCL9* and *CXCL10* were higher in anti-Jo-1 antibody-positive DM-ILD patients than in those with idiopathic pulmonary fibrosis, which distinguished this disease entity from idiopathic pulmonary fibrosis and anti-signal recognition particle antibody-positive myositis (Richards et al., 2009). Oda et al. reported that serum levels of *CXCL10* of DM-ILD patients at 2 weeks after treatment initiation were significantly higher in the death group, and the change ratio of serum levels of *CXCL10* and *CXCL11* was higher in the death group, suggesting that serum levels of *CXCL10* may be possible biomarkers of disease activity and prognosis in DM-ILD (Oda et al., 2017). However, the role of other genes such as *PLAUR* in DM-ILD has not been reported. PLAUR overexpression in DM-ILD patients than healthy control samples was validated by RT-qPCR in our study. The mechanism how the genes participate in the etiology and development of DM-ILD requires further investigation.

AS events have been studied in many diseases, such as cancer (Kahles et al., 2018), autoimmune disease (Aravilli et al., 2021), and diabetes (Newman et al., 2017). However, the mechanisms of AS in DM-ILD remained unexplored. Here, we present a systemic analysis of AS in DM-ILD and found 866 differentially expressed AS events, including SE, A3SS, A5SS, MXE, and RI. Moreover, one gene had one or more AS events. To further explore the role of AS, we overlapped 35 genes with the parent genes of differentially expressed AS events and identified *PLAUR* as the key gene in AS-dependent DM-ILD. Zhang et al. reported that *PLAUR* had indirect connections with miR-155 and miR-146a, which are the top-ranked miRNAs responding to cystic fibrosis plasma (Zhang et al., 2019b). *PLAUR*, *IL4*, *COL3A1*, *CXCL8*, *MMP2*, *LOX*, *MMP14*, *COL1A2*, and *PLOD2* were also found to be slightly upregulated in alveolar fibroelastosis and in organizing pneumonia but downregulated in non-specific interstitial pneumonia and usual interstitial pneumonia (Jonigk et al., 2019). Increased uPAR expression, as seen in the bronchial epithelium of patients with asthma, leads to attenuated wound repair which may contribute to the development and progression of airway remodeling in asthma (Stewart et al., 2012). In the current study, *PLAUR* had interactions with multiple candidate key genes and was found to participate in complement and coagulation cascades, neutrophil-associated immune response, and coagulation-related biological responses. Although the exact function of DM-ILD remains unclear, *PLAUR* has been shown to be associated with autoimmune disease (Göbel et al., 2016; Kobayashi et al., 2021) and relates to pulmonary dysfunction. Thus, *PLAUR* may be involved in the pathogenesis of DM-ILD through immune-mediated mechanism.

Based on the aforementioned results, we hypothesized that immune cell distribution of the disease group and the control group might be different. We found more neutrophils and less naive B cells in DM-ILD patients *via* CIBERSORT analysis. A strong positive correlation was observed between the expression of *PLAUR* and the abundance of neutrophils. The important role of neutrophils in DM-ILD was confirmed again, suggesting that *PLAUR* may regulate DM-ILD through neutrophil-related immune response. There are some limitations in our study. We identified *PLAUR* between DM-ILD patients and normal control blood samples. Further studies will be needed to explore the expression of *PLAUR* in DM-ILD patients and DM patients without ILD complications and in other sample types such as pulmonary tissue. Moreover, the detailed function of *PLAUR* in DM needs to be studied in future *in vivo* and *in vitro* experiments.

## Conclusion

In conclusion, *BDKRB2*, *CAMP*, *COL1A1*, *F2RL1*, *F3*, *MS4A2*, *CXCR3*, *ICAM1*, *IGFBP3*, *IGHE*, *IL1A*, *IL1B*, *IL1RN*, *IL6*, *CXCL8*, *IL9R*, *IL10*, *IL18*, *CXCL10*, *KRT18*, *LAMA5*, *LAMC2*, *LEP*, *MET*, *CXCL9*, *MMP1*, *MMP2*, *MMP9*, *PLAUR*, *PPARG*, *RNASE2*, *RNASE3*, *SOD2*, *TLR4*, and *IL1RL1*, which were associated with inflammation and immunization, were identified as key genes in DM-ILD. Moreover, the present study is the first to systematically analyze AS events in patients with DM-ILD and find the association of *PLAUR* expression with DM-ILD. Furthermore, we preliminarily explore the possible mechanisms such as neutrophil-related immune response in DM-ILD. These findings enrich our understanding of DM-ILD, which may benefit DM-ILD patients in future.

## Data Availability

The datasets presented in this study can be found in online repositories. The names of the repository/repositories and accession number(s) can be found in the article/Supplementary Material.
