# Supplementary material for: Transcriptome Sequencing Identifies PLAUR as an Important Player in Patients With Dermatomyositis-Associated Interstitial Lung Disease
Source: Front Genet. 2021 Dec 6;12:784215. doi: 10.3389/fgene.2021.784215 (PMC8685457; doi:10.3389/fgene.2021.784215)
Supplement: Supplementary file 1 [file DataSheet3.zip › Supplementary Material S3/Clean data.docx]

| Sample | Length | ReadS | QC30(%) | Qc% |
| --- | --- | --- | --- | --- |
| LR20L26DX196 | 36-141 | **23864193*2** | **100%** | 51 |
| LR20L26DX197 | 36-141 | **21055419*2** | **100%** | 50 |
| LR20L26DX198 | 36-141 | **21247101*2** | **100%** | 50 |
| LR20L26DX199 | 36-141 | **20736445*2** | **100%** | 50 |
| LR20L26DX200 | 36-141 | **24511779*2** | **100%** | **51** |
| LR20L26DX201 | 36-141 | **22354280*2** | **100%** | **51** |
| LR20L26DX202 | 36-141 | **22582670*2** | **100%** | **50** |
| LR20L26DX203 | 36-141 | **17475041*2** | **100%** | **51** |
| LR20L26DX204 | 36-141 | **18558805*2** | **100%** | **51** |
| LR20L26DX205 | 36-141 | **23276483*2** | **100%** | **50** |
| LR20L26DX206 | 36-141 | **24780030*2** | **100%** | **50** |
| LR20L26DX207 | 36-141 | **25180365*2** | **100%** | **51** |
