# Supplementary material for: Transcriptome Sequencing Identifies PLAUR as an Important Player in Patients With Dermatomyositis-Associated Interstitial Lung Disease
Source: Front Genet. 2021 Dec 6;12:784215. doi: 10.3389/fgene.2021.784215 (PMC8685457; doi:10.3389/fgene.2021.784215)
Supplement: Supplementary file 2 [file Table1.DOCX]

Table S1. Demographic of DM-ILD patients and healthy controls

| variables | DM-ILD patients  (RNA-sequencing n=6) | Healthy controls (n=6) | *P*  value | DM-ILD patients (RT-qPCR n= 10) | Healthy controls  (n=10) | *P* value |
| --- | --- | --- | --- | --- | --- | --- |
| Age, years | 48.3 ± 8.4 | 42.0 ± 12.5 | 0.33 | 52.2 ± 10.4 | 49.4 ± 8.8 | 0.45 |
| Gender | 5F / 1M | 5F / 1M | 1.00 | 6F / 4M | 7F / 3M | 1.00 |

F, Female; M, Male
