# Supplementary material for: Transcriptome Sequencing Identifies PLAUR as an Important Player in Patients With Dermatomyositis-Associated Interstitial Lung Disease
Source: Front Genet. 2021 Dec 6;12:784215. doi: 10.3389/fgene.2021.784215 (PMC8685457; doi:10.3389/fgene.2021.784215)
Supplement: Supplementary file 3 [file DataSheet4.zip › Supplementary Material S4/Mapping result.docx]

| Sample | LTotal_Reads  （QC-passed reads + QC-failed reads） | Total_Mapped | paired in sequencing | properly paired |
| --- | --- | --- | --- | --- |
| LR20L26DX196 | **51214179** | **49190989(96.05%)** | **47728386** | **39597912(82.97%)** |
| LR20L26DX197 | **45537673** | **44455941(97.62%)** | **42110838** | **34575198(82.11%)** |
| LR20L26DX198 | **45461159** | **44346649(97.55%)** | **42494202** | **34543908(81.29%)** |
| LR20L26DX199 | **45939975** | **44896076(97.73%)** | **41472890** | **33354172(80.42%)** |
| LR20L26DX200 | **54403331** | **52922913(97.28%)** | **49023558** | **40392726(82.39%)** |
| LR20L26DX201 | **48512216** | **47428809(97.77% )** | **44708560** | **35508118(79.42% )** |
| LR20L26DX202 | **48525253** | **47352453(97.58% )** | **45165340** | **36204812(80.16% )** |
| LR20L26DX203 | **37945708** | **36953194(97.38% )** | **34950082** | **28747476(82.25%)** |
| LR20L26DX204 | **40258608** | **39117732(97.17%)** | **37117610** | **30315640(81.67%)** |
| LR20L26DX205 | **57502506** | **56293622(97.90%)** | **46552966** | **40313680(86.60% )** |
| LR20L26DX206 | **55405241** | **54210727(97.84%)** | **49560060** | **42961148(86.69% )** |
| LR20L26DX207 | **59074166** | **57246440(96.91%)** | **50360730** | **42345790(84.08%)** |
