# Supplementary figures and images for: Transcriptome Sequencing Identifies PLAUR as an Important Player in Patients With Dermatomyositis-Associated Interstitial Lung Disease
Source: Front Genet. 2021 Dec 6;12:784215. doi: 10.3389/fgene.2021.784215 (PMC8685457; doi:10.3389/fgene.2021.784215)

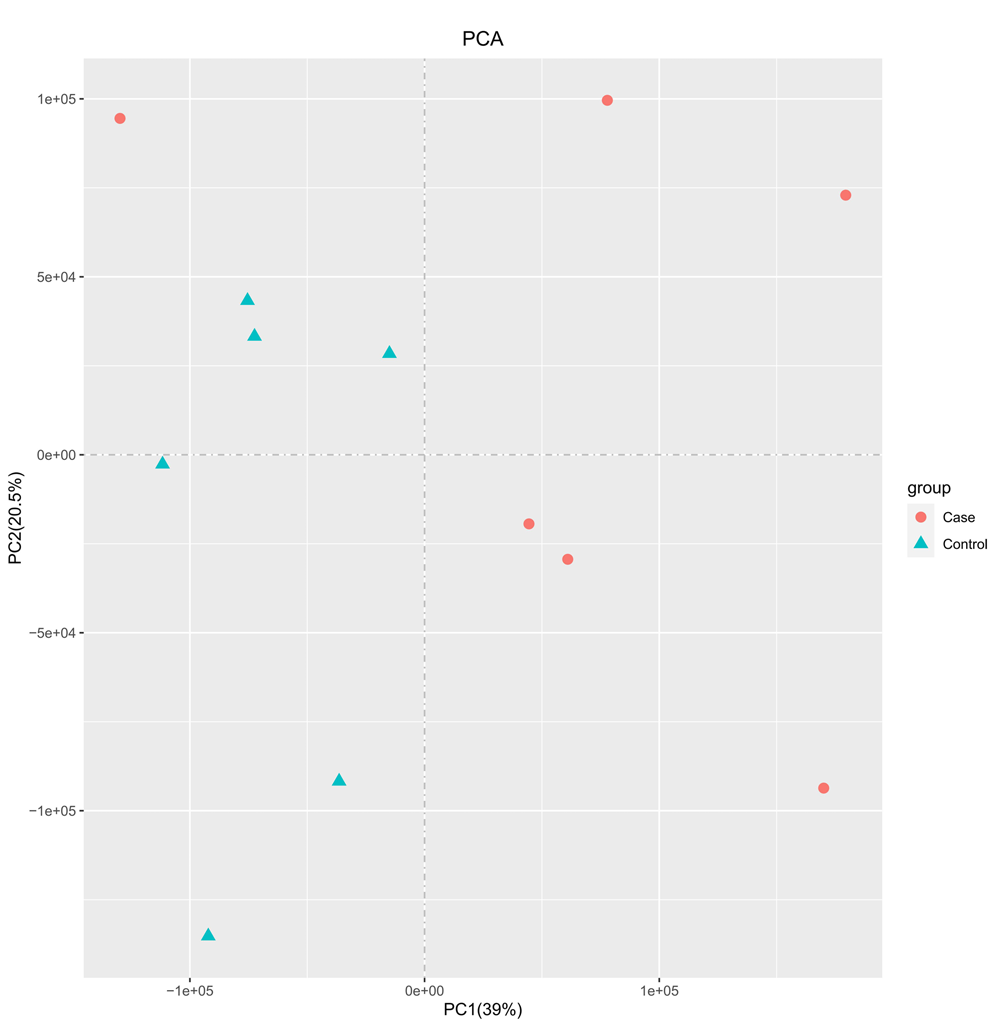

Supplement: Supplementary file 6 [file Image1.TIF]

# Boxplot of immuneCell

CellType

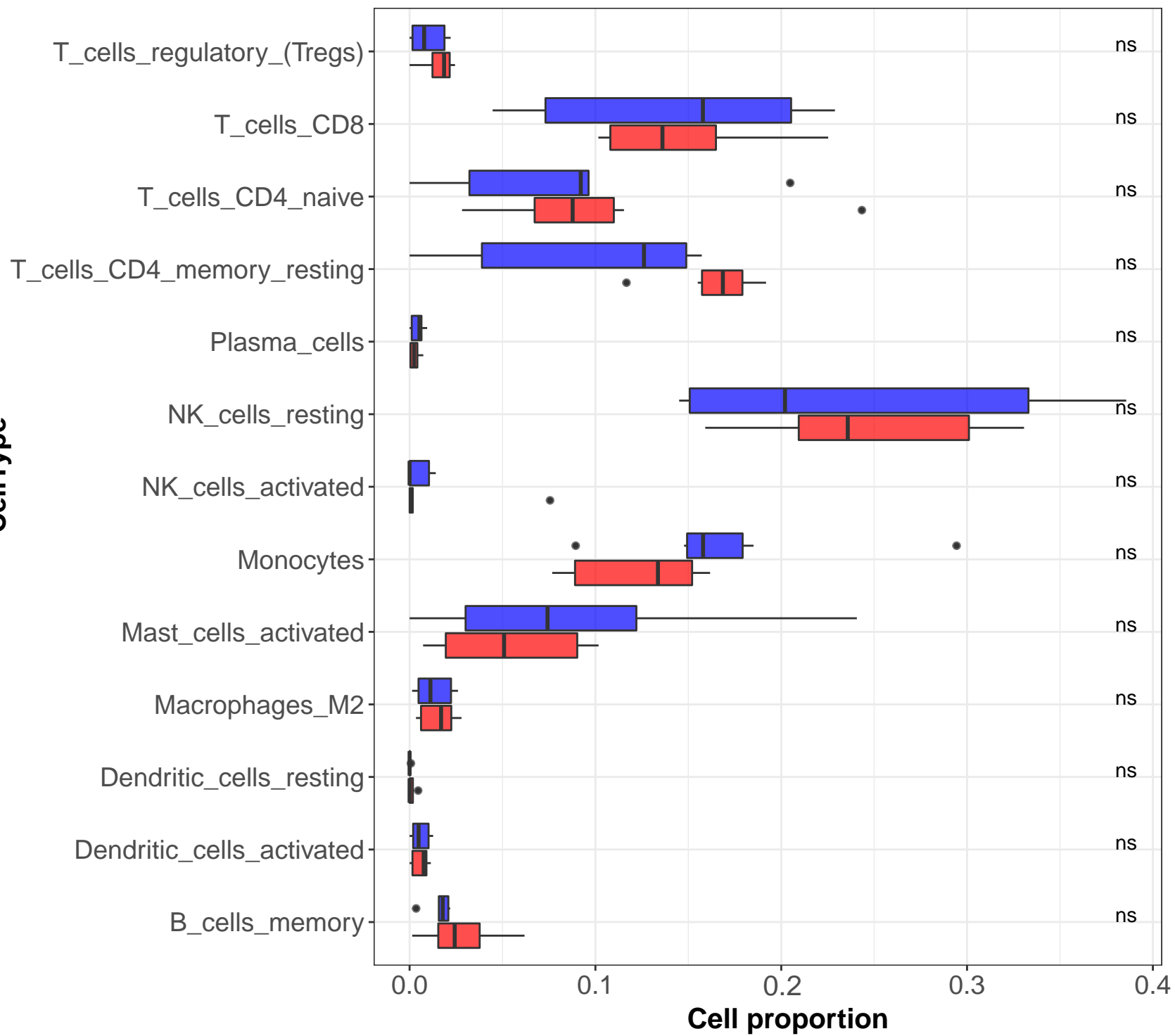

Supplement: Supplementary file 7 [file DataSheet1.PDF]
