# Supplementary material for: Transcriptome Sequencing Identifies PLAUR as an Important Player in Patients With Dermatomyositis-Associated Interstitial Lung Disease
Source: Front Genet. 2021 Dec 6;12:784215. doi: 10.3389/fgene.2021.784215 (PMC8685457; doi:10.3389/fgene.2021.784215)
Supplement: Supplementary file 8 [file DataSheet2.zip › Supplementary Material S2/LR20L26DX197.R2.paired_fastqc.html]

LR20L26DX197.R2.paired.fastq.gz FastQC Report 

FastQC Report

星期六 30 一月 2021  
LR20L26DX197.R2.paired.fastq.gz

## Summary

- Basic Statistics
- Per base sequence quality
- Per tile sequence quality
- Per sequence quality scores
- Per base sequence content
- Per sequence GC content
- Per base N content
- Sequence Length Distribution
- Sequence Duplication Levels
- Overrepresented sequences
- Adapter Content

## Basic Statistics

| Measure | Value |
| --- | --- |
| Filename | LR20L26DX197.R2.paired.fastq.gz |
| File type | Conventional base calls |
| Encoding | Sanger / Illumina 1.9 |
| Total Sequences | 21055419 |
| Sequences flagged as poor quality | 0 |
| Sequence length | 36-141 |
| %GC | 50 |

## Per base sequence quality

## Per tile sequence quality

## Per sequence quality scores

## Per base sequence content

## Per sequence GC content

## Per base N content

## Sequence Length Distribution

## Sequence Duplication Levels

## Overrepresented sequences

No overrepresented sequences

## Adapter Content

Produced by FastQC (version 0.11.9)
