# Supplementary material for: Transcriptome Sequencing Identifies PLAUR as an Important Player in Patients With Dermatomyositis-Associated Interstitial Lung Disease
Source: Front Genet. 2021 Dec 6;12:784215. doi: 10.3389/fgene.2021.784215 (PMC8685457; doi:10.3389/fgene.2021.784215)
Supplement: Supplementary file 10 [file Table2.DOC]

GeneAS_typeORC3A3SSELP4A3SSNAGPAA3SSZNF767PA3SSPPP1R12BA3SSHECTD1A3SSBACE1A3SSZNF717A3SSMSH5-SAPCD1A3SSATAD2A3SSCLCN6A3SSSLC66A2A3SSZNF286AA3SSRPS6KA4A3SSSETDB1A3SSSECISBP2A3SSCELF1A3SSHSPBP1A3SSRICTORA3SSWDR75A3SSBMP1A3SSUSP19A3SSZNF467A3SSMETTL26A3SSTECRA3SSTGFAA3SSMPV17A3SSFTSJ1A3SSDNAH1A3SSPI4KAP2A3SSRIC3A3SSADAM8A3SSCD1EA3SSPPP6R3A3SSSLC29A2A3SSLARGE2A3SSALS2CLA3SSPHF20L1A3SSIMPA1A3SSCYB561A3SSS100PBPA3SSNR1H2A3SSC1orf159A3SSLGALS9BA3SSSLC35B1A3SSTIMM17BA3SSTMEM147-AS1A3SSPSIP1A3SSPSMD2A3SSSPSB2A3SSSPAG7A3SSTMEM106AA3SSSH3TC1A3SSASIC3A3SSALPK1A3SSAP4E1A3SSMTFR1LA3SSSLC25A39A3SSTRIM8A3SSNKIRAS2A3SSPPT2-EGFL8A3SSPROCA1A3SSC1orf109A3SSCCDC142A3SSCYP2R1A3SSCERS4A3SSDYRK1BA3SSPMS2A3SSL3MBTL1A3SSZNF461A3SSTMEM218A3SS A5SS RI SESNHG17A3SS MEX SEU2AF1L4A3SS RI SECD27-AS1A3SS SEMT1EA3SS SEBIRC5A3SS SELAIR1A3SS SEMYO15BA3SS SEHDAC8A3SS SEMMABA3SS SEFAM114A2A3SS SERAD51CA3SS SESETD5A3SS SELARP4A3SS SEAPTXA3SS SEPANK4A3SS SEGORASP1A3SS SETNFRSF25A3SS SENME6A3SS MEXCUL7A3SS MEXWDR45A3SS RIAMTA3SS RIASGR2A5SS SEPCBP1-AS1A5SS SEEPB41L2A5SS SEHMOX2A5SS SECARD8A5SS SETCF3A5SS SEGSTZ1A5SS SER3HDM1A5SS SEPFKMA5SS SEPTCH1A5SS SESYTL3A5SS SEEPORA5SS SEZNF862A5SS SEN4BP2L2A5SS MEXING4A5SS RIMGAMMEX SEMCCC1MEX SEFAM214AMEX SEPOLR1CMEX SESLC37A3MEX SETANGO2MEX SEHACL1MEX SEFYB1MEX SEMTHFSDMEX SEPOGZMEX SECAMK2DMEX SEPOMT1MEX SEDPP8MEX SECTNND1MEX SEATXN2MEX SEUBE3AMEX SEAHSA2PMEX SESCMH1MEX SETULP3MEX SEPRKCQ-AS1MEX SEFAM228BMEX SEPCNX1MEX SEVNN3MEX SENEMFRI SEFAM219AA5SSMAP4A5SSKLHDC4A5SSCD1DA5SSIFI27L1A5SSGOLGB1A5SSNUDT1A5SSPAQR6A5SSDOK3A5SSMGME1A5SSTTLL4A5SSWASH2PA5SSUSP32A5SSLIG1A5SSHDAC9A5SSAFG3L1PA5SSMRRFA5SSIL32A5SSFAXDC2A5SSRABGAP1LA5SSASPSCR1A5SSULK3A5SSPIDD1A5SSCEP164A5SSCHTF18A5SSAC005562.1A5SSALG9A5SSALMS1A5SSZNF37AA5SSMRPL11A5SSWDR26A5SSCASKA5SSCAMK2GA5SSCREB3L4A5SSCPT2A5SSBTAF1A5SSMX2A5SSVDRSESLMAPSEVPS29SEIFT52SEPTPN23SEGAPVD1SEWASHC4SEGFM2SEAL162231.2SEMACF1SESOS1SENUP155SEZNF664SEBTBD3SEAC087500.1SEPARP15SELINC01128SEKANSL2SEAP000646.1SETREX2SERFWD3SEPCED1BSEPKIGSESHISAL2ASEZSCAN9SEIP6K2SEIKBIPSEMETTL23SEPTPRSSECOQ6SEIZUMO4SERCOR1SEPOLISECHD9SEFCGRTSERO60SEPAPOLASESLC22A4SEAATKSEAL157935.3SENRMSEIGF2BP2SESLC15A2SETUG1SEARL1SEBBC3SEMFSD6SEIKZF5SESMARCD1SEZFYVE1SEDUS2SEANKMY1SEKDM7ASESRSF7SELONRF3SEZNF182SETBCELSEZNF619SEBCL2L11SEAL355385.2SESTK36SEACYP2SELYRM1SETOP3BSEPKD2SECEP290SEMRM1SELOXL2SECCDC66SETASOR2SEC10orf88BSEARHGEF12SENMRK1SESNRNP70SEALDOCSERCHY1SEDCP1ASEPRDM2SESLC16A6SEDMXL2SEDCAF6SEAL451136.1SENEIL1SEKIAA1958SESPOPSELRRC23SEMFFSEPABPC1LSESTAU2SEZNF808SEMTRRSEPOLLSEDICER1SENCOR2SECLASP1SEWDR59SEAC010522.1SEDMTNSECCDC18-AS1SEMYO19SEDCUN1D4SERBM23SECDK8SECREB1SEQDPRSETRIM41SEPPP2R3BSESRPK2SEARNTSETASORSEGARS1-DTSESLC22A15SEHSPA14SEABCA1SEMGAT1SELLGL2SEALDH8A1SELRP8SEMCUSEZNF211SETPCN1SECADSESKP2SESRSF11SEINTS9SEAC093157.1SERWDD4SEEXO5SESH2B2SESFI1SEKIFBPSESLC43A1SEPLEKHB2SERAB3ASEZNF692SESRSF1SEPPP4R1LSEPRORPSETOMM34SEFNDC3ASEANAPC16SEZNF71SEPCK2SETBL1XR1SEMRE11SEMYEF2SEBFARSETAF2SEC1QTNF3-AMACRSECAPRIN2SESTARD4SEZSCAN32SEZFAND4SEIRF3SEATRIPSENBPF8SEFGD4SELGALS9SETTI1SETRNT1SESNX25SEZNF81SEZMYND8SETMBIM6SEGIHCGSEANKRD49SEPATL2SECCDC91SEZNF707SEKDM6ASEIL6STSECRYZSETARBP2SECABIN1SEITSN2SECCT6P1SEGUF1SEGUSBSEISOC2SECAPN12SESGMS2SEWDR27SEPARLSEPOC5SEGRNSECDAN1SESPECC1L-ADORA2ASENQO2SERBMS2SEDRAM2SECEP192SERNF111SEMASTLSEASB3SEKANSL3SEAPOBEC3BSESTAU1SELINC02284SEDBF4SEDNHD1SEPTPN2SEIFNLR1SEMIDEASSETOM1L2SESIMC1SEFAM210ASELIN9SEAFMIDSENFS1SEZNF180SECBX6SEPBRM1SEFRG1HPSECROCCP2SEERCC6SETTC21BSETAMM41SENEDD8SETMEM14BSEMSMO1SEZFYVE16SESYNJ2BPSEUNC5CLSEPRH1SEHPSESYCP2SEENO3SEPROSER1SEASPHSEPKIASENAB1SEC1orf112SEPARGSEZNF100SEZNF92SEKIF13BSEUIMC1SEACCSSESUGP2SEPRPF39SEASAP2SEPAX8-AS1SESP3SEGBF1SEGOSR2SESLC7A7SETPT1-AS1SESLC25A10SESLFN11SESTK19SESLC2A8SEPEAK1SELTO1SEPRR14LSEPLA2G6SERALGAPA2SECAMKK1SECUL4BSEIFNGR1SEDPEP2SEMFSD14CSEGDE1SEZNF417SESIRT2SEFANCASETTC3SEPHYKPLSEMAP3K8SEPCBP3SEALG13SEEFCAB14SEATP8B4SEDDX60LSEPLEKHA3SERNF121SEANKRD46SECYBC1SELRRC42SEAC093752.1SELDLRSETOMM40LSEZNF384SECFAP44SEMPHOSPH9SEWBP1SEZMYM5SECTRLSEPAK4SEZNF586SECLDND1SEFAM219BSEZC3H4SESAMD9LSENDUFA3SEPDPRSEPRKAR1ASETEDC1SESH2B1SEMESDSEPIGGSEMUC20-OT1SELINC02785SEAL022318.4SEZBTB8OSSEGPR141SEXRRA1SEXIAPSEPCSK5SESLC36A4SEPER1SESLC29A3SEDAGLBSENNTSETCP11L2SETM2D1SERNMTSEMICAL1SEZHX1SEMROH6SESIPA1L1SEFBXO5SEANKRD17SEHPS5SEELF4SEDTX2P1SETCEA3SEAGPAT4SECAPN15SEPPCDCSEZKSCAN3SEZNF789SETDRD7SEUBFD1SELINC00174SECXorf65SEADAMTS10SEITPRIPL1SETMEM107SELINC00667SEKIAA0232SETLK2SEELL2SEPOLR3ESEGPR157SEMTERF4SEFAM107BSEC1QASEGALNT7SEPPIELSERIPOR2SEELMO3SEZNF542PSECES4ASEPOLR3GLSECASTOR3SENDOR1SECDK16SELINC-PINTSEWDYHV1SEMKS1SERBM12BSETRIM59SELOXHD1SESGMS1SEZNF195SENARS2SEARMC10SEDHX58SEDNAJB14SES100A6SELRCH3SEFBXO28SEANKRD55SECOG8SETRMT2BSEWASH6PSEERVK13-1SEMCTP2SEIRAK4SETNPO2SEMT1XSEQTRT2SEAC096667.1SEMGRN1SESNHG14SESULT1B1SEAC011899.2SEPLAURSESCAPSESESTD1SEPOLR3DSESLC35E2BSENIPA2SEEEF1AKMT1SEARID2SEBRAFSEIQCB1SESPATS2SEMIATNBSECOA1SEMBD2SEC21orf58SELRRC14SEKCTD9SESCML4SESLC9B2SEZNF611SEAC118553.2SESUSD1SEHIPK1SESRP9SEPISDSECXorf38SEMKRN2SEAC068580.4SES100A13SEZNF17SERPP38SERCBTB2SEZNF280DSEVPS8SEPOLR2DSECDK10SESPASTSELETMD1SEAGO3SEZBTB17SEVPS13BSEDCLRE1CSEACER3SESF1SEMRNIPSERERESEBIN1SEFANCD2SEMCTP1SEGBA2SEPDIA5SEWDR47SEVPS33AMEXGPHNMEXODF2MEXPOLR3AMEXFGRMEXTRMT61BMEXZNF276MEXDNAJA2MEXACY1MEXTLR10MEXCYRIBMEXCLEC7AMEXYEATS2MEXRPAINMEXANKRD36MEXDHDDSMEXINTS8MEXRSKRMEXGTPBP10MEXUXS1MEXCOLGALT2MEXSHOC2MEXBICRALMEXMSRAMEXJPXMEXCTBP2MEXGSNMEXATP6V0A1MEXRAD51DMEXSLC35E3MEXADCY4MEXCAPN10MEXNF1MEXPLB1MEXATXN3MEXFASTKMEXABCC1MEXKANSL1MEXNPIPA1MEXSIN3AMEXNOL4LMEXOBSCNMEXMDM1MEXZNF266MEXN4BP2L1MEXINO80MEXMIGA1MEXRESF1MEXSTK17BMEXSRSF4MEXFLVCR2MEXGOLGA8BMEXDLG1RIFAN1RIHMG20BRITTC31RIRIPOR1RIMPPE1RIRMC1RIATP5MC1RIDUS3LRISLAIN2RIIRF5RICTPS1RIPSMB4RITLE2RITRMT6RIRASGRP4RIHAUS5RIRPS2RINUP54RINPRL2RICOQ8BRIGALERIHTRA2RIUNC13DRIMARS1RIHLA-ARIRAD52RIMDP1RISPG7RIDPY19L3RIWDR13RICD72RICCDC14RIATG16L1RIRNPC3RIEIF3GRISNHG29RIFMNL1RI
